# Esophageal Stent Migration Diagnosed With Point-of-Care Ultrasound

**DOI:** 10.7759/cureus.49418

**Published:** 2023-11-26

**Authors:** Samuel Harris, A. Brad Hall, Charlotte Derr

**Affiliations:** 1 Emergency Medicine, University of South Florida Morsani College of Medicine, Tampa, USA; 2 Emergency Medicine, Lakeland Regional Health, Lakeland, USA

**Keywords:** esophageal stent, abdominal imaging, stomach, emergency medicine physician, chest pain, abdominal pain, point-of-care ultrasound, ultrasound, sonography, esophageal stent migration

## Abstract

This unique case depicts the first published report of a physician using point-of-care ultrasound to diagnose an esophageal stent migration. Discussed in this article are the sonographic findings that clinicians should be familiar with when evaluating patients with abdominal pain or chest pain who have a history of an esophageal stent. When coupled with a high index of suspicion, ultrasound can be one of the most portable, readily available, low-cost, and minimally invasive techniques for making a rapid diagnosis of esophageal stent migration.

## Introduction

Esophageal self-expanding stents are used for a variety of pathologies including unresectable esophageal malignancies, refractory esophageal strictures, and management of perforations and fistulas. After placement, migration represents a common complication and may occur in up to approximately one-third of cases [1]. Often esophageal stent migration remains asymptomatic; however, patients may experience returning dysphagia, chest pain, abdominal pain, and intestinal obstruction or perforation [2]. We present the first reported case of point-of-care ultrasound (POCUS) used to diagnose a migrated esophageal stent in a patient who presented to the emergency department (ED) for chest and abdominal pain. POCUS is an increasingly popular area of advancement in healthcare worldwide. Providers from numerous specialties can improve healthcare costs and efficiency by utilizing ultrasound at the bedside to obtain and interpret images in order to reach a diagnosis [3]. In some cases, X-ray may be used to quickly diagnose migrated esophageal stent, but POCUS has broader utility in the assessment of undifferentiated symptoms, and can be used to rapidly assess for emergent complications of stent migration.

## Case presentation

A 63-year-old male with a past medical history of chronic obstructive pulmonary disease, hepatitis C, and three myocardial infarctions (not currently on any medications) presented to the ED via emergency medical services with the initial concern of midsternal, non-radiating chest pain that woke him up from sleep. The chest pain quickly resolved after arrival at the ED; however, he continued to complain of intermittent, non-radiating, severe abdominal pain located in the epigastric region. He had generalized, intermittent abdominal pain over the preceding two weeks which acutely worsened over the previous two days. He had one episode of vomiting after presentation to the ED, but otherwise had no associated symptoms including fever, chills, shortness of breath, change in bowel habits, or urinary concerns. After initial treatment with fentanyl, his pain briefly improved from 10 out of 10 to 6 out of 10. The pain quickly returned and was refractory to further analgesic treatment including high dose opiates and sub-dissociative dose ketamine. He endorsed a history of “back surgery” of unknown type and a tonsillectomy. He reported a 60-pack-year smoking history and daily ethanol use; however, he had not drunk any alcohol for the last week due to his abdominal pain. He endorsed a remote history of cocaine use and intravenous drug use.

His presenting vital signs were a blood pressure of 137/98 mmHg, heart rate of 100 beats per minute, respiratory rate of 19 breaths per minute, a temperature of 97.8 F, and oxygen saturation of 100% on room air. The patient’s cardiopulmonary exam was unremarkable. His abdomen was soft, and non-distended, with normal bowel sounds. Significant tenderness was present upon palpation of the epigastric region and mid-abdomen, and guarding was present. The patient was in obvious acute distress secondary to abdominal pain, ill-appearing, and anxious.

**Figure 1:**
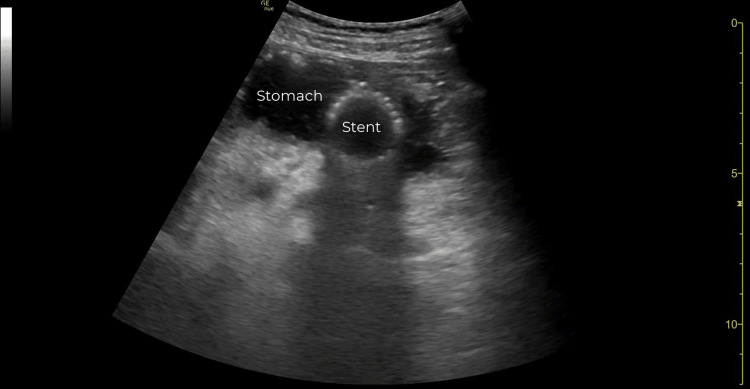
Point-of-care ultrasound of the stomach demonstrating a 2.41 cm x 2.51 cm round, echogenic, and fenestrated structure (short-axis view).

**Figure 2:**
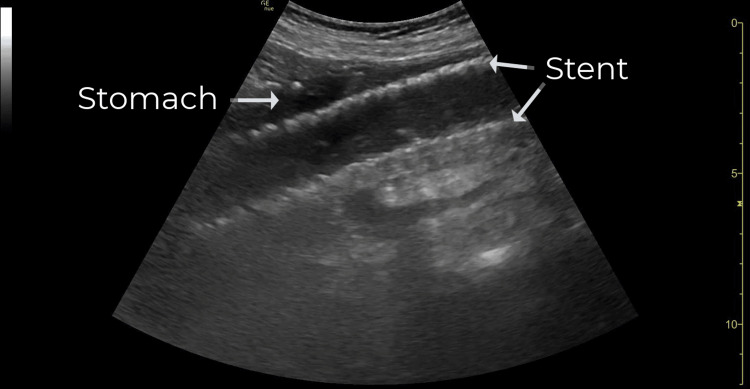
Point-of-care ultrasound of the stomach demonstrating a cylindrical form that was greater than 10 cm in length, echogenic, and fenestrated (long-axis view).

Due to his original chief complaint of chest pain and his current abdominal pain, the attending emergency physician was concerned about an aortic emergency such as aortic aneurysm or dissection. The POCUS examination of the abdominal aorta was initiated by the emergency physician using a curvilinear transducer placed in the epigastric region in the transverse orientation. During the examination, the abdominal aorta was noted to be unremarkable. A fluid-filled stomach containing a 2.41 cm x 2.51 cm round, echogenic, fenestrated structure was seen just anterior to the abdominal aorta (Figure 1). As the transducer was rotated 90 degrees the echogenic structure elongated into a cylindrical form that was greater than 10 cm in length (Figure 2). The lumen of the form contained fluid that appeared to flow freely between the form and the stomach (Video 1).

**Video 1:** Point-of-care ultrasound of the stomach demonstrating a cylindrical form that was greater than 10 cm in length, echogenic, and fenestrated (long-axis view).

The ultrasound characteristics of the foreign body were suggestive of a synthetic graft or large stent. Shortly after the POCUS, the patient was asked again about any past abdominal surgeries and was specifically asked if he had ever had an endoscopy. At that point the patient was able to recall possibly having had an endoscopy; however, he was unable to provide any further history of timing, reason for the procedure, history of esophageal malignancy, or other gastrointestinal pathology.

**Figure 3:**
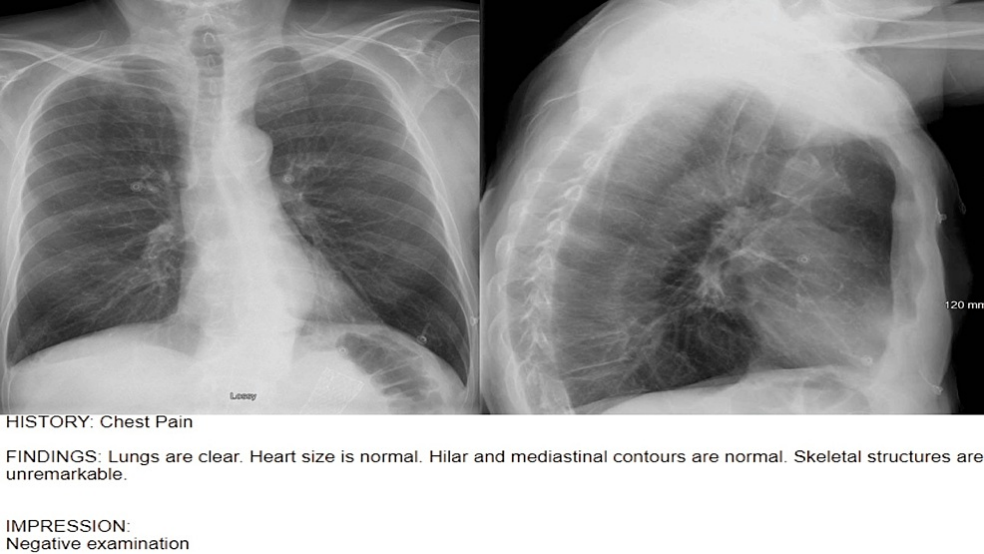
PA and lateral CXR were interpreted as negative examination by the radiologist.

Laboratory testing revealed a leukocytosis of 14,800 cells/mm^3^, with a neutrophilic predominance. The remainder of the complete blood count, comprehensive metabolic panel, lactate, lipase, and urinalysis were unremarkable. The initial troponin was within normal limits. Initial EKG showed normal sinus rhythm without ischemic changes. The chest X-ray was read by the radiologist as negative for any acute cardiopulmonary abnormalities (Figure 3).

**Figure 4:**
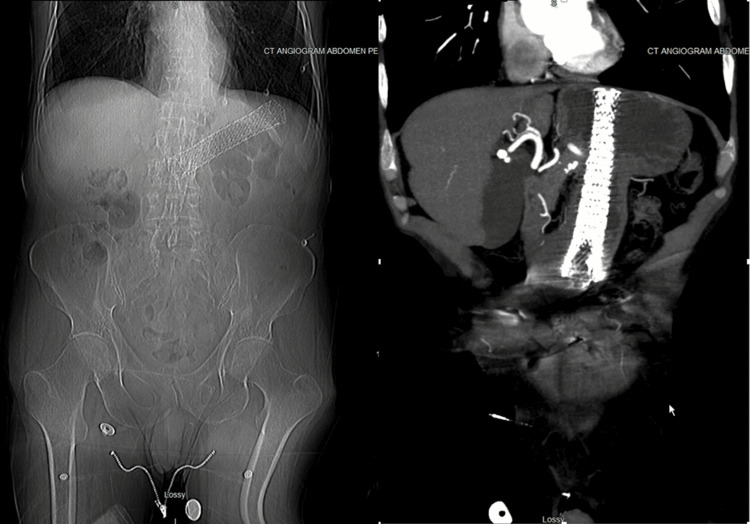
CT scout image and CT angiogram of abdomen and pelvis with malpositioned esophageal stent within the stomach.

The findings on POCUS suggested stent migration, which led the ED physician to request an expedited CT scan of the abdomen and pelvis. No abdominal X-ray was obtained prior to the definitive imaging of the CT scan, as the diagnosis of esophageal stent migration was already made using POCUS. The CT demonstrated mass-like thickening of the distal esophagus and a malpositioned esophageal stent entirely within the stomach (Figure 4).

**Figure 5:**
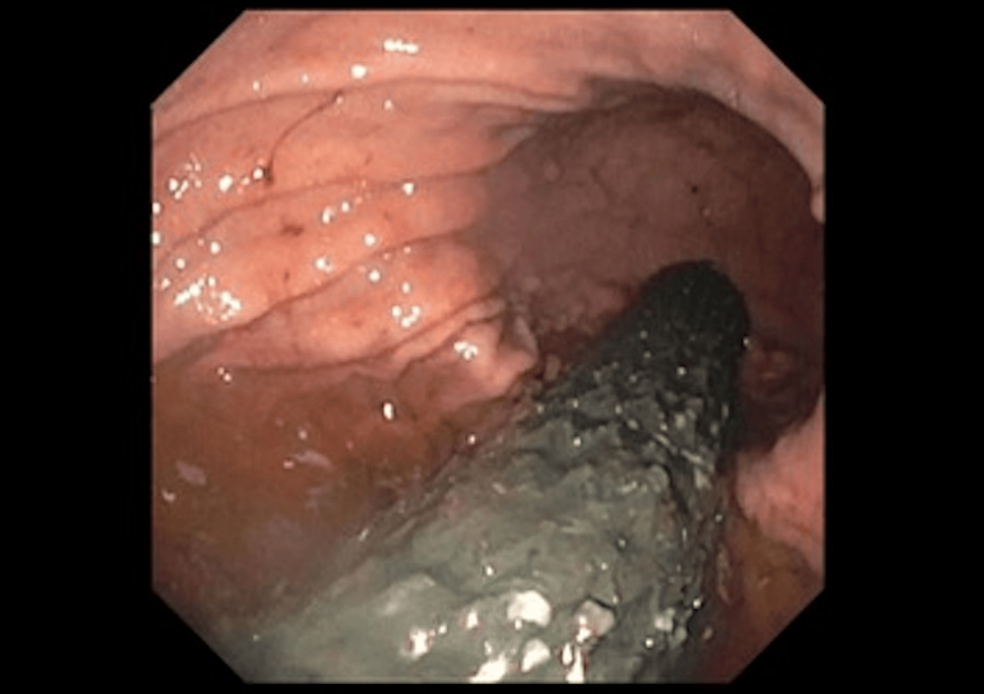
Esophagogastroduodenoscopy (EGD) with migrated esophageal stent within the gastric body.

Once the CT was visualized by the ED physician, an emergent consult was placed to gastroenterology for further management recommendations. Gastroenterology performed an esophagogastroduodenoscopy the following day and found a benign-appearing severe esophageal stenosis which was dilated to 8 mm, severe esophagitis without bleeding, an esophageal ulcer, and a migrated esophageal stent within the gastric body (Figure 5).

**Figure 6:**
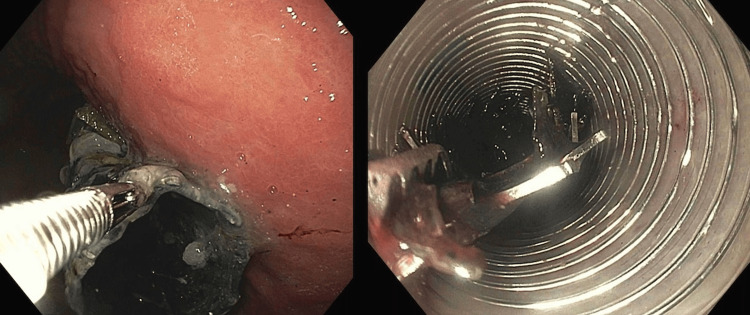
Esophagogastroduodenoscopy (EGD) with successful esophageal stent removal.

The esophageal stent was unable to be retrieved due to the degree of proximal stenosis. The stricture was at the level of the mid esophagus 13 cm proximal to the lower esophageal sphincter. A repeat EGD was performed one week later with serial dilation of the stricture and successful stent removal. The stent was removed following stricture dilation using rat-tooth forceps visualized by an endoscope which was retracted through an overtube to protect the airway and esophagus during removal (Figure 6). Biopsies performed at a later date showed only esophagitis without evidence of mass or cancer. Records were unable to be obtained in regard to the original reason for stent placement, but it was likely placed due to dysphagia secondary to severe stenosis. The hospital course was complicated by difficult to control likely multifactorial abdominal pain. The patient was discharged three days following stent removal with outpatient GI follow-up for management of gastritis, esophagitis, and stricture.

## Discussion

Esophageal stent migration is a common complication and carries a risk of intestinal obstruction and perforation. Removal of migrated stents is recommended urgently rather than emergently and is usually safe with a high success rate [4,5]. Our case involved a 63-year-old male patient presenting with severe intractable abdominal pain who provided an unreliable history. Prompt use of bedside ultrasound led to an answer for the etiology of his symptoms within minutes. A search of the literature revealed that this is the first report of an emergency physician performing POCUS in the diagnosis of esophageal stent migration, and is the first article to describe the appearance of a migrated stent on ultrasound.

POCUS is a well-established tool for the evaluation of undifferentiated abdominal pain. POCUS can shorten the time to diagnosis for many emergent causes of abdominal pain such as abdominal aortic aneurysm, appendicitis, cholecystitis, appendicitis, diverticulitis, bowel obstruction, and ovarian torsion [6]. In addition to these more established techniques, the use of perioperative gastric POCUS to evaluate aspiration risk is an evolving diagnostic tool, which may be used in the qualification and quantification of gastric contents prior to intubation [7]. Gastric ultrasound may also be used to assess for proper gastric tube positioning and can aid in the diagnosis of gastric peptic ulcer disease [8,9].

A stent on ultrasound will appear as a hyperechoic, fenestrated, cylindrical structure. It may be seen in the gastric body, fundus, or small bowel. In the event that a stent is visualized in the subdiaphragmatic enteric system, it can be assumed that it has migrated. A properly positioned esophageal stent will not be amenable to ultrasound of the abdominal cavity.

If esophageal stent migration is suspected, POCUS can be used to quickly identify the stent and assess for life-threatening sequelae of this complication including perforation and obstruction [6]. The identification of these feared complications would make stent migration an emergency rather than an urgency. Intraperitoneal free air noted on ultrasound could help expedite the mobilization of resources for definitive surgical management thus reducing morbidity and mortality.

## Conclusions

For the patient in this case with poor medical insight who was unable to provide an accurate account of his medical history, surgical history, and recent symptoms the initial differential diagnosis was very broad. Early use of abdominal POCUS led to a quicker diagnosis and consultation with specialists for definitive care. We were also able to rule out abdominal aortic pathology which helped to guide the decision of the most appropriate additional imaging. While our patient would most likely have had a CT based upon the initial presenting symptoms of chest pain and abdominal pain, the POCUS enabled the ED physician to quickly gain an understanding of the etiology of the patient’s severe pain in order to expedite management and order appropriate testing. Without the use of POCUS alternative imaging may have been pursued including CT chest dissection or a formal right upper quadrant ultrasound, which would not have been as useful in making the diagnosis.

While the radiologist interpreted the chest radiograph as normal, it should be noted that there was evidence of stent migration identified at the inferior-most portion of the posterior-anterior image. This revelation occurred subsequent to the diagnosis of stent migration made through POCUS. This underscores the importance of reviewing the radiographs you order, serving as a valuable reminder for clinicians to actively engage in the diagnostic process.

Ultrasound is a valuable tool in the workup of undifferentiated abdominal pain. It is cost-effective, does not require the use of ionizing radiation, and can be performed rapidly at the bedside. In stable patients for whom a stent migration is suspected CT should be considered when the ultrasound is inconclusive and may be beneficial for operative planning.
